# Visual-spatial working memory in ADHD: new evidence for a storage rather than a processing deficit

**DOI:** 10.1007/s00787-026-02987-8

**Published:** 2026-02-23

**Authors:** A. Coclici, D. Canu, J. Bretthauer, M. Biscaldi, N. Smyrnis, Christoph Klein

**Affiliations:** 1 Clinic for Child and Adolescent Psychiatry, Medical Centre, University of Freiburg, Freiburg im Breisgau, Germany; 2 Department of Child and Adolescent Psychiatry, Medical Faculty, University of Cologne, Cologne, Germany; 3 2nd Psychiatry Department, Medical School, National and Kapodistrian University of Athens, University General Hospital “Attikon”, Athens, Greece

**Keywords:** ADHD, Eye tracking, Visual-spatial working memory, Anti-saccade task, Memory-guided saccade task

## Abstract

The ability to voluntarily control eye movements is part of the “executive function” domain, which includes working memory and is impaired in neuro-developmental disorders such as ADHD. The present study aimed to investigate two types of “executive” saccades – anti-saccades (AS) and memory-guided saccades (MGS) – as well as their relationships in samples of children and adolescents with ADHD and neurotypical controls. To that end, anti-saccades and memory-guided saccades were recorded in n = 65 participants with ADHD (age 9.2–17.1 years) and n = 74 neurotypical controls (aged 9.1–17.3 years) as part of a larger ocular-motor test battery, using the EyeLink 1000 + eye tracker (SR-Research). Results reveal less correct MGS with lower spatial accuracy as well as a trend for less correct AS with normal spatial accuracy in participants with ADHD, compared to neurotypical controls. Furthermore, the spatial accuracies of AS and MGS were significantly correlated in the control (r=.35) and ADHD (r=.26) groups, corresponding to medium effect sizes (eta2: control: 0.12, ADHD: 0.07). These findings suggest that the storage of spatial information in working memory, as required in the MGS task, is impaired in ADHD, while the processing of this information, as required in the AS task, remains largely intact. The correlations between the two task parameters in both groups confirm that storage and processing are related abilities in working memory and that this relationship is basically intact in ADHD.

## Introduction

Attention-Deficit/Hyperactivity Disorder (ADHD) is defined by impaired attention and hyperactivity / impulsivity and figures among the most prevalent disorders of childhood and adolescence. It is considered a clinically and aetiologically heterogeneous disorder, including different subtypes, causes and outcomes [8, 32]. In addition to the clinical manifestations, ADHD is characterized by impaired *executive functions* (EF; [18, 21]), which are considered as core to the pathology of the disorder. Working memory, as part of the executive function system, enables processing of information stored on the short-term (“working with memory”), thus comprises storage and processing and is typically categorized into verbal/numerical versus visual/spatial information [3]. Deficits in working memory are among the most consistent findings in the cognitive-behavioral ADHD literature [42]. According to the meta-analysis conducted by Martinussen et al., [29], it is in particular the storage and the executive/processing component of *visual-spatial* working memory that is impaired in ADHD. A more detailed and nuanced understanding of working memory impairments in ADHD would be of critical importance, not only from a pathophysiological perspective, but also given its potential role as a therapeutic target [24, 26] or diagnostic endophenotype [4].

Studies that have been conducted to measure *visual-spatial* working memory deficits in ADHD have used a variety of paradigms [25, 29], predominantly employing tasks that assess memory capacity via coarse recall-based motor responses, imposing comparatively high memory load, and not providing an isolated measure of spatial processing due to task-inherent confounding of storage and processing demands [9, 34] or the absence of a genuine processing component [11]. In contrast, the present study uses eye-tracking paradigms with small memory set sizes and immediate spatial manipulation (see below) to obtain precise and direct measures of spatial accuracy and processing speed, thereby minimizing storage-related contributions and enabling a differentiated assessment of visuospatial processing, storage, and their interrelation across development in ADHD and control participants.

Two tasks from the eye-tracking domain are potentially useful in studying visual-spatial working memory in that they enable the quantification of the *spatial accuracy* of working memory in addition to reaction time or accuracy (defined as: proportion correct-to-incorrect), the most commonly provided information from working memory tasks with manual responses. These tasks are the anti-saccade (AS) and the memory-guided saccade (MGS) tasks. In these tasks, participants are instructed to inhibit a saccade to a peripherally presented stimulus and either look straight in the (horizontal) *mirror-image position* of that stimulus (AS), or to the *remembered position* of that stimulus a few seconds after its offset (MGS). “Mirror-image position” and “remembered position”, hence, constitute the commonality of AS and MGS, which is the absence of a visible stimulus as the saccade landing point. This common feature stands in stark contrast to real-life saccades, which are almost always directed at visible objects, beings etc.

Specifically, in the *AS task*, the process of determining the horizontal mirror-image position of a stimulus presented to the left or right of central fixation is called “vector inversion” [16]. Vector inversion requires a precise spatial representation of the to-be-spatially-inverted target position but no storage of it (over time) as it occurs immediately. In the *MGS task*, by contrast, directing the landing point of the saccade to the position of a previously shown but no longer visible dot requires its precise spatial representation in working memory and the storage of that representation over time; it does not require spatial inversion of a saccade vector. As both AS and MGS thus rely on internally generated landing points, both types of saccades have been considered as “volitional” or “*endogenously generated*” saccades [17, 33]. Given that storage and processing are inter-related sub-functions within working memory [3], such generic terminology for AS and MGS can be justified at a theoretical level. One way to address this topic empirically is by studying how inter-individual differences in these tasks co-vary.

It should be noted, however, that while spatial accuracy is one of the standard parameters of the MGS task, the spatial accuracy of AS has been barely investigated in the experimental and clinical literature. Consequently, there is to our knowledge no information about the relationship between these two aspects of visual-spatial working memory and how that relationship potentially differs from normal in conditions with WM impairments, such as ADHD.

Based on these considerations, the aims of the present study are as follows: (1) compare patients and controls in AS and MGS performance employing precise measurement of spatial accuracy; (2) determine the statistical relationship (correlation) of AS and MGS task performance – in particular: spatial accuracy – as an index of construct validity both in controls and in patients; (3) determine how these measures of spatial accuracy and their relationship change with age. We hypothesized deficits in participants with ADHD compared to controls in both AS and MGS, substantial positive correlations between the parameters of these tasks and improvements with increasing age. Conjointly, these findings would indicate that AS and MGS task performances draw (at least partially) from the same underlying ability, may be assigned to the same construct and indicate spatial working memory impairments in ADHD. Given that reaction time and accuracy of AS and MGS in ADHD have received clearly more attention in the literature than spatial accuracy, this last-mentioned aspect of task performance will constitute the focus of this report.

## Methods

The present study was approved by the Ethics Committee of the University of Freiburg (EK 124/17; PI CK) and all participants or their caregiver gave their informed written consent.

### Participants

After the exclusion of participants for technical reasons (see data analysis below), the study sample included *n* = 139 participants, of which *n* = 65 had an ADHD diagnosis and *n* = 74 were neuro-typically developing, recruited through the departmental data base, for patients: if they fulfilled ICD-10 diagnostic criteria for ADHD (F90.0/1; ascertained from expert clinicians via anamnestic interviews, behavioural observations, and Conners’ parent and teacher rating scales), but did not present with the following co-morbidities, Autism Spectrum Disorder (F84), Schizophrenia or paranoid disorder (F20-F29) and for controls: if they had no psychiatric or neurological diagnoses. For all participants, exclusion criteria were IQ < 70, neurological disorders in the past and at present as well as eye-sight impairments (unless corrected). ADHD patients on stimulant medication (*n* = 20) refrained from taking these drugs for > 24 h before testing. For this sample, the power for the detection of a medium effect of d = 0.5 at alpha=0.05, one-tailed, was 0.90 (G*Power 3.1.9.6; Faul, 2020).

The ADHD sample was aged 13.1±2.4 years (range: 9;2–17;1ys), had an average IQ of 101±12 (range: 75–136), and included 25 females (38.5%). Neurotypical controls were aged 14.1±1.8 years (range: 9;1–17;3ys), had an average IQ of 120.1±17.4 (range: 73–160), and included 41 females (55.4%). Controls were somewhat older than patients (t = 2.48, *p* = .015), included more females (t = 2.01, *p* = .046) and scored higher in IQ (t = 7.66, *p*<.001).

### Procedure

Individual testing sessions took place in the departmental eye tracking lab (sound-attenuated Faraday cage), lasted for about 150–180 min, comprised of overall 11 different eye movement tasks (order counterbalanced) and included breaks after every task. Stimuli were generated with the software package Experimental Builder (SR-Research, ON, Canada). Brightness within the Faraday cage was measured (Peaktech 5035) and kept constant at 70-80Lx.

For the *Memory-Guided Saccade* task (“MGS”) an initial central fixation point was presented for 1,000ms, followed by an additional red circle presented horizontally for 100ms at one of the following pseudo-randomly sequenced positions to the left or right (±2.5°, ±5.0°, ±7.5°, ±10.0°, ±12.5°, ±15.0°) of fixation. After a memorization gap of 2,500-4,500ms, the central fixation cross disappeared, and after another 1,500ms the red circle re-appeared and stayed on screen for 1,000ms. After an inter-trial interval (ITI) of 1,000ms, the next trial began. Participants were instructed to remain with their gaze on the central fixation, remember the peripheral position of the red circle and look at this position upon the disappearance of the central fixation. Six practice trials preceded 48 test trials.

For the *Anti-Saccade* task (“AS”), each trial began with the presentation of a central fixation cross, followed 1,000ms later by a green circle presented in pseudo-random order at 7° to the left or right of fixation and remaining there for 1,000ms. After an ITI of 1,000ms, the next trial began. During the overlap condition, central fixation stayed on while the peripheral cue was visible; during the gap condition, central fixation was set off 200ms before the onset of the cue. Participants were instructed to look at the central fixation cross and look straight to the opposite-site mirror-image position of the cue upon its appearance (see [14]). Eight practice trials preceded 100 test trials.

The IQ-testing constituted a second session with groups of up to 8 participants and employed the CFT-20R [40].

### Apparatus

Eye movements were recorded with the EyeLink1000 + eye tracker (SR-Research, ON, Canada), employing a 1000Hz sampling rate with 0.01 degrees resolution. Stimuli were presented on a 24’’ LCD-screen (1920*1080 pixels, 60 Hz refresh rate, viewing distance 90 cm). A 9-point-calibration preceded each task and subsequent drift corrections were accomplished as required. Recordings were recalibrated whenever necessary.

### Data analysis

Primary data analysis (parametrisation) was accomplished for only one eye per participant, namely the one with better technical data quality according to the test protocols. Only saccades faster than 25°/s and larger than 1° were analysed, excluding micro-saccades [22]. Trials with eye lid closures or head movements were excluded. Only horizontal saccades were included.

*Spatial inaccuracy* of correct AS and correct MGS was defined as *percentage* *error in* *amplitude* (“PEA”) according to Mostofsky et al., [30]: PEA = 100*│1 – (eccentricity of final saccadic landing point [°] / eccentricity of target stimulus [°])│.

The first up to three saccades directed at the target (primary saccade plus two corrective saccades) were considered to derive the PEA measures. In about 57–63%, the first saccade reached a position closest to the target; in about 31–35% of the trials, this position was reached with the second and in 5–8% with the third saccade. This grossly summarized result held for both groups (AS: all ps>0.08; MGS: all ps>0.22) and both tasks (see minima/maxima above).

*Reaction time* was defined as the time between stimulus presentation and saccade onset for AS and as the time between the disappearance of the central fixation cross and saccade onset for MGS. For the derivation of *correct* AS, only saccades with reaction times 131–1000 ms post cue-onset were used.

*Direction errors* in the AS task were primary saccades with regular reaction times directed towards the cue. *Accuracy* was defined as the proportion of correct AS and MGS. At least 20 or 18 correct AS or MGS, respectively, were required for a participant to be included in the data analysis [5, 10].

A total of 12 subjects (*N* = 8 ADHD group; *N* = 4 control group) had to be excluded from the initial sample of 147 subjects (*N* = 73 ADHD group; *N* = 74 control group). Three subjects from the ADHD group were excluded due to missing data for one or both tasks due to discontinuation or non-performance. Another three subjects from the ADHD group were excluded for not reaching the specified minimum number of 20 anti-saccade trials (20%). As part of the statistical outlier adjustment (one and a half times the interquartile range), 4 subjects (*N* = 1 ADHD group; *N* = 3 control group) were excluded for MGS and 2 subjects (*N* = 1 ADHD group; *N* = 1 control group) were excluded for AS.

ANCOVAs used GROUP (ADHD vs. control) as a between-subject factor and included age, sex and IQ as co-variates. Leaving out sex and IQ as co-variates did not principally alter the results; leaving out age reduced between-group differences slightly. Furthermore, we correlated AS and MGS parameters co-varying for age, and determined their relationship with age (employing univariate linear regression). Finally, in order to assess whether the correlation between the spatial accuracies of AS and MGS changes with increasing age, we correlated the individual products of z-scores for AS and MGS (z_AS_ * z_MGS_) with participants’ ages (see [20]). A zero correlation of this z-score product and age would suggest age-invariance of the relationship between AS and MGS spatial accuracy. Two-tailed *p*-values are reported throughout.

## Results

1. *Group differences.* Comparing the ADHD and neurotypical control groups and including age, sex and IQ as co-variates in the ANCOVA models revealed that participants with ADHD made significantly *fewer* correct MGS than controls (*F1, 134* = 4.034, *p* = .047, *η*² = 0.029) and exhibited significantly *less* spatial accuracy of correct MGS (*F1, 134* = 14.78, *p* < .001, *η*² = 0.099; see Fig. 1). Reaction times of correct MGS did not differ significantly between groups (*F1, 134* = 0.681, *p* = .411, *η*² = 0.005). By contrast, spatial accuracy of anti-saccades did not differ between patients and controls (*F1, 134*=0.06, *p*=.815, *η*²=0.001) and participants with ADHD exhibited almost the same proportion of correct AS (*F1, 134*=0.455, *p*=.501, *η*²=0.003) and initiated these with roughly the same reaction time (*F1, 134*=0.419, *p*=.519, *η*²=0.003) compared to controls.

**Fig. 1:**
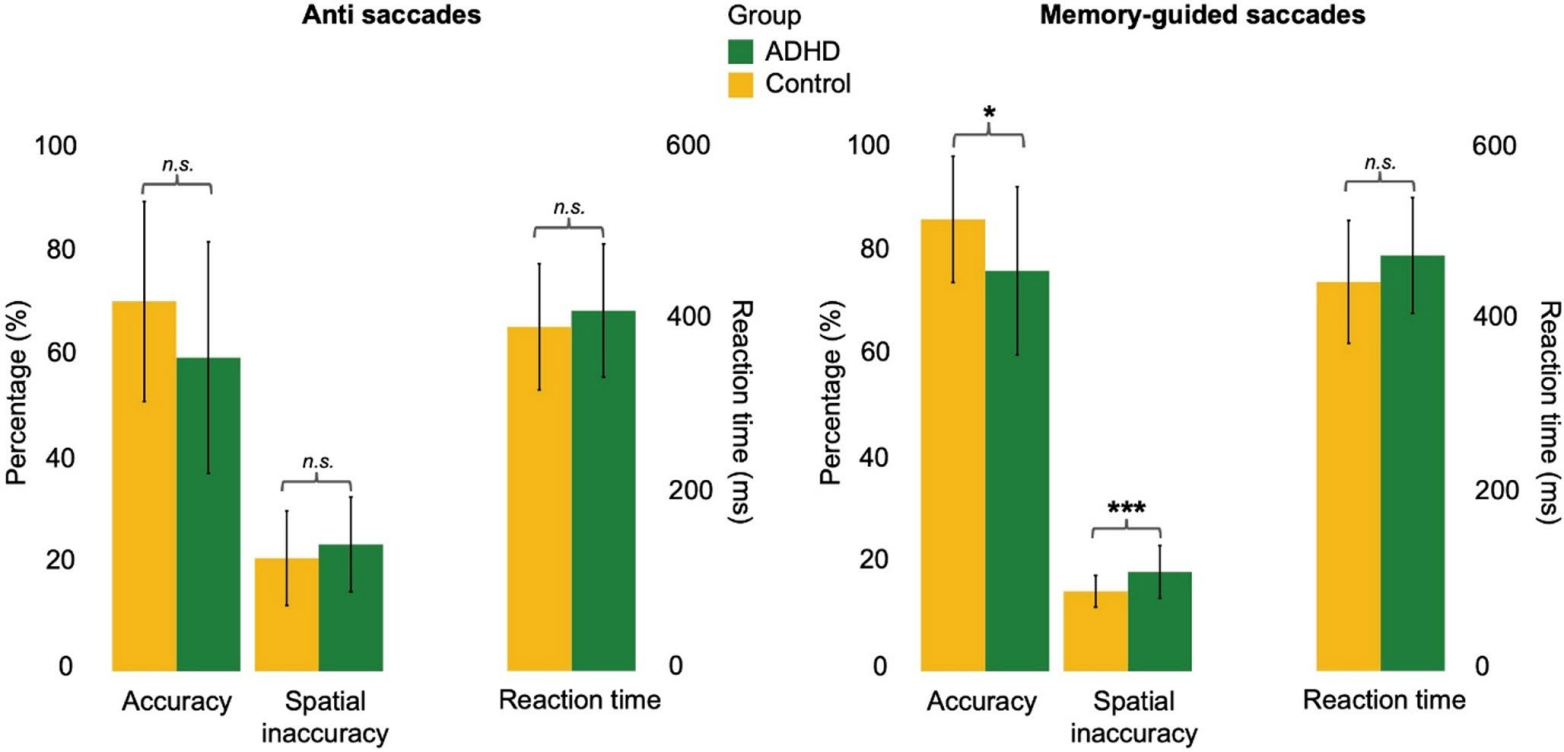
Group differences in the anti-saccade and memory-guided saccade tasks. Mean accuracy (%), spatial inaccuracy (%) and reaction time (ms) for correct memory guided saccades (MGS) and correct anti saccades (AS) for patients (green) and controls (yellow). Bars represent group means, error bars indicate standard deviations. Accuracy and spatial inaccuracy are displayed on the left y-axis, while reaction time is plotted on the right y-axis. Asterisks indicate significant group differences with age, sex and IQ included as co-variates in the ANCOVA-models: *p*<.05 (*), *p*<.01 (**), *p*<.001 (***)
2. *Correlations between AS and MGS task parameters*. Correlations between the *spatial accuracies* of correct AS and MGS showed substantial and significant correlations both within the ADHD group (*r*=.40; *p*=.001, eta2=0.16) and the neurotypical control group (*r*=.38; *p*=.001, eta2=0.14; see Fig. 2c). Age correlated with both measures (see Fig. 2a, b), so these correlations dropped after co-varying for age (ADHD: *r*=.26; *p*=.037, eta2=0.07; control: *r*=.35; *p*=.003, eta2=0.12). Likewise, *reaction times* of correct AS and MGS were correlated by *r*=.29 (*p*=.018, eta2=0.08) in patients and *r*=.60 (*p*<.001, eta2=0.36) in controls (*r*=.20, *p*=.119, eta2=0.04 and *r*=.58(*p*<.001, eta2=0.35) in patients and controls when co-varying for age, respectively). Similarly, AS and MGS *accuracies* (proportions of correct responses) were correlated by *r*=.68 (*p*<.001, eta2=0.46) in patients and *r*=.39 (*p*=.001, eta2=0.15) in controls (*r*=.62, *p*<.001, eta2=0.38 and *r*=.42(*p*<.001, eta2=0.18) in patients and controls when co-varying for age, respectively). Co-varying additionally for sex and IQ did not further change these coefficients. Furthermore, z-score correlations for AS or MGS spatial accuracy were un-correlated with age (controls: − 0.03; patients: 0.03, both *p*s>0.80), suggesting that in our sample, the linear relationship between these two measures of spatial accuracy remains stable between ages 9 and 17 years (age-invariance).

**Fig. 2:**
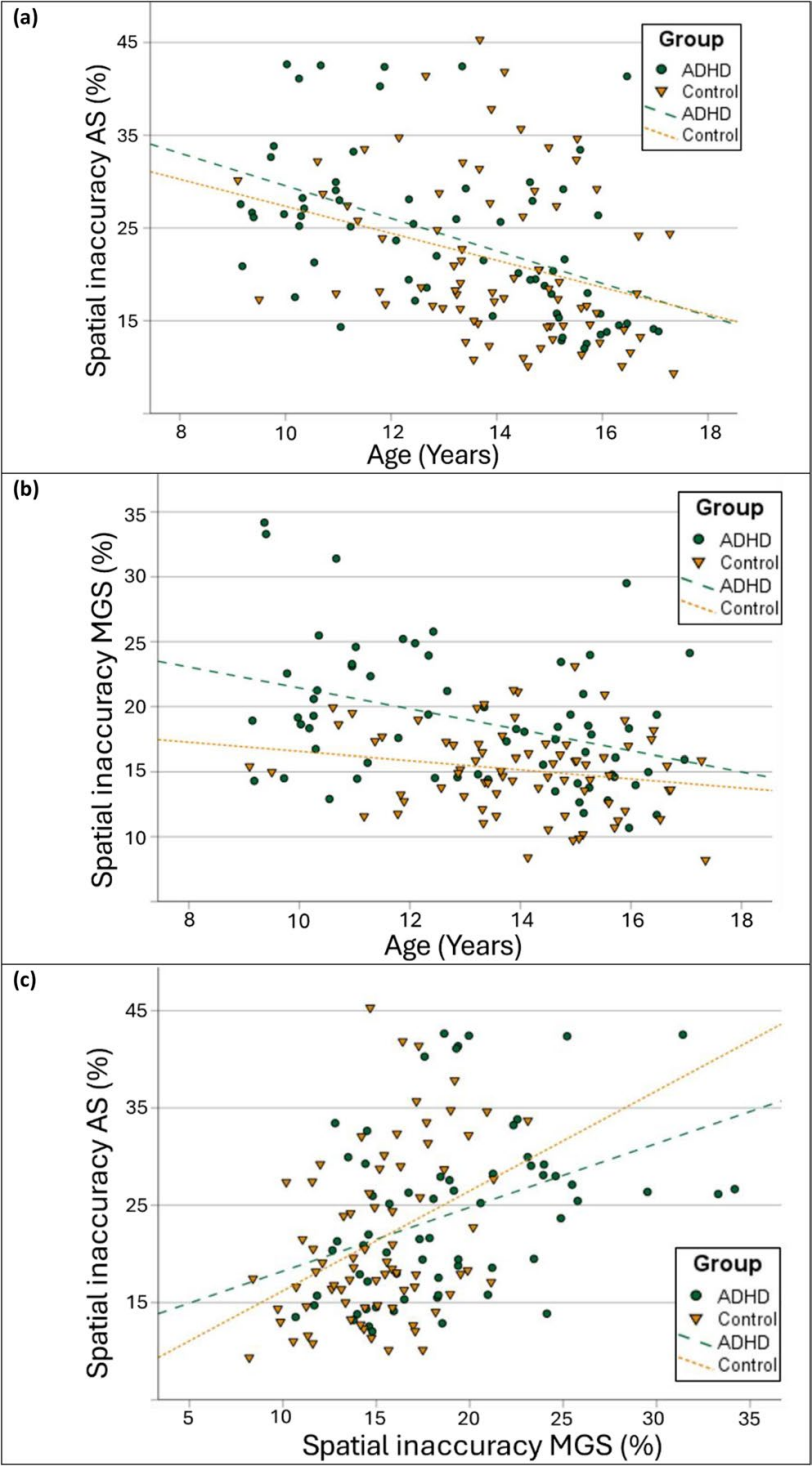
Relationship between spatial inaccuracy in antisaccade and memory-guided saccade tasks with age and across tasks. (**a**, **b**) Scatter plots for the spatial inaccuracy (%) of (**a**) correct antisaccades (AS) and (**b**) correct memory-guided saccades (MGS) as a function of age (years), and (**c**) the relationship between AS and MGS spatial inaccuracy (%), shown separately for patients and controls (ADHD: green circles, dashed linear regression line; controls: yellow triangles, dotted linear regression line)
3. *Correlations with age*. Figure 2a and b reveal that the spatial accuracy of both AS and MGS improved with increasing age. This improvement was descriptively somewhat greater in patients than controls.

## Discussion

The present study set out to the hypotheses, (1) that participants with ADHD show impaired spatial accuracy of both AS and MGS; (2) that AS and MGS task parameters are significantly correlated in either group; and (3) that both spatial accuracies improve with age. While hypothesis (1) could be confirmed for MGS only, hypotheses (2) and (3) could be confirmed fully.

1. D*Group differences in spatial accuracy*. Regarding the spatial accuracy of AS, Loe et al., [27] reported no differences between patients and controls, Goto et al., [12] found differences only in younger participants (6–8 years) and Hakvoort Schwerdtfeger et al., ) and [13] found no differences in adults with ADHD. In the AS task, the endogenous definition of the saccade landing point is accomplished through vector inversion, which requires a spatial representation of the target position but does not require its storage as in the MGS task. Given the normal spatial accuracy of AS in participants with ADHD, we conclude that the endogenous definition of the saccade landing point through vector inversion, an executive component of visual-spatial working memory [38], is intact in ADHD.].]. However, regarding the normal spatial accuracy of anti-saccades in the present study, this impairment of the central executive could be limited to processes with this sort of continuous attention control, leaving its functionality in immediate responses as manipulation of spatial information intact.
2. *Correlations between AS and MGS task parameters*. The three different task parameters analysed here correlated in different strengths between tasks, with spatial accuracy showing across groups the lowest and medium-sized and accuracy the highest and large-sized correlations. To our knowledge, correlations between AS and MGS spatial accuracies have not been reported before. They could plausibly result from what is common to both tasks regarding the achievement of spatial accuracy, the endogenous determination of the saccade landing point, which requires in the case of both tasks some involvement of visual-spatial working memory [38]. This requirement is manipulation of a spatial position for AS and storage of a spatial position for MGS. The present findings therefore confirm the often-reported correlation of storage and processing in working memory as a constituent feature of this functional system [6]. The fact that in our study the correlation between AS and MGS spatial accuracies were only slightly lower in ADHD compared to controls – but still in the range of medium-sized effects – indicate that the interaction between storage and processing is basically intact in our patient group. That only storage (MGS) but not processing (AS) was impaired in patients is not incommensurate with this conclusion. Finally, the z-score correlations for AS and MGS spatial accuracy were uncorrelated with age. This suggests that the discriminant validity of spatial accuracy as a WM sub-construct can be considered as developmentally invariant, despite its massive improvements during late childhood and adolescence (see next discussion point).
3. *Correlations with age.* That both AS and MGS spatial accuracy improved with increasing age across late childhood and adolescence in the ADHD and control groups in similar manners not only aligns with the known improvement of ocular-motor functions during childhood and adolescence [19, 28], it also speaks in favour of similar developmental trajectories of the measured parameters in participants with ADHD and controls [21].

### Further findings

Our data analysis of AS and MGS spatial accuracy has shown in both groups that neither the first [5] nor the last [28] anti- or memory-guided saccade alone is sufficient in quantifying spatial accuracy in these tasks. This result argues strongly for deriving AS or MGS spatial accuracy measures from the spatially closest of the primary, secondary or tertiary saccades towards the remembered or the inverted target position in these tasks. Furthermore, this study did not reveal any differences between participants with ADHD and controls regarding anti-saccade task accuracy. While the literature is not entirely consistent with studies often lacking statistical power [35], our study is not the first that did not show significant differences between these groups [1, 15, 39].

### Conclusions

The results of the present study confirm that the spatial accuracy of correct anti-saccades contains valid information that can be substantially correlated with the spatial accuracy of memory-guided saccades. As participants with ADHD exhibit normal spatial accuracy of anti-saccades it may be concluded that the inversion of the saccade vector in AS tasks, a processing component of working memory, is intact in this group. The reduced spatial accuracy of memory-guided saccades, by contrast, suggests deficient storage of the spatial target position in visual-spatial working memory. As both measures of spatial accuracy were substantially correlated in either group, we conclude that association between storage and processing in visuo-spatial working memory is intact in ADHD. An early meta-analysis on effect sizes of group differences in different aspects of working memory found Cohen’s d-values ranging between 0.43 and 1.06 [29]. The group effect sizes reported here score in the lower part of that range for the MGS task (0.35≤d≤0.66) and are close to zero for the AS task (d≤0.11). Given that group differences in short-term recall may decline with declining cognitive load [31], the minimal cognitive load imposed by our tasks may account for the low to medium effect sizes that we found. Using age as a co-variate in studies with children and adolescents with ADHD is of great importance to unveil potential differential developmental effects in those with ADHD (developmental decelerations, developmental delays etc., manifesting in GROUP x AGE interactions). Ideally, the study of differential developmental effects should be based on age-matched groups.

In summary, our findings provide the first evidence in the ADHD literature that a fundamental aspect of visuospatial processing - spatial remapping as an index of central executive functioning - is preserved in ADHD when memory load is minimized. This result could not be demonstrated in previous studies due to the confounding of executive processing and storage or the absence of genuine executive demands under high memory load. Moreover, the presence of comparable, age-invariant correlations between measures of visuospatial storage and executive processing in ADHD and control groups suggests a preserved and developmentally stable functional architecture of visuospatial working memory in ADHD.

## Data Availability

Data can be made available upon request to the corresponding author.
